# Hepatitis B Virus X Protein Sensitizes TRAIL-Induced Hepatocyte Apoptosis by Inhibiting the E3 Ubiquitin Ligase A20

**DOI:** 10.1371/journal.pone.0127329

**Published:** 2015-05-20

**Authors:** Hang Zhang, Changxin Huang, Yan Wang, Zhe Lu, Ningtong Zhuang, Dongjiu Zhao, Jianqin He, Liyun Shi

**Affiliations:** 1 Department of Basic Medical Science, Key Lab of Immunology and Molecular Medicine, School of Medicine, Hangzhou Normal University, Hangzhou, Zhejiang 310036, China; 2 Department of Oncology, Affiliated Hospital of School of Medicine, Hangzhou Normal University, Hangzhou, Zhejiang 310036, China; 3 Department of Infectious Diseases, First Affiliated Hospital, School of Medicine, Zhejiang University, Hangzhou, Zhejiang 310003, China; UMR INSERM U866, FRANCE

## Abstract

Hepatitis B virus (HBV) infection causes hepatocyte death and liver damage, which may eventually lead to cirrhosis and liver cancer. Hepatitis B virus X protein (HBx) is a key antigen that is critically involved in HBV-associated liver diseases. However, the molecular basis for its pathogenesis, particularly in liver damage, has not been well defined. Herein, we report that HBx was able to enhance the susceptibility of hepatocytes to TNF-related apoptosis-inducing ligand (TRAIL)-induced apoptosis. Increased sensitivity to TRAIL was associated with HBx-induced upregulation of miR-125a, which, in turn, suppressed the expression of its putative target gene, A20 E3 ligase. Importantly, we demonstrate that the defective expression of A20 impaired the K63-linked polyubiquitination of caspase-8, which reciprocally enhanced the activation of caspase-8, the recruitment of Fas-associated death domain (FADD), and the formation of death-inducing signaling complex (DISC), thereby promoting HBx-mediated apoptotic signaling. Accordingly, antagonizing miR-125a or ectopically expressing A20 in hepatocytes abolished the pro-apoptotic effect of HBx. Conversely, the overexpression of miR-125a or knockdown of A20 mimicked HBx to enhance TRAIL susceptibility in hepatocytes. Thus, we establish, for the first time, a miR-125a/A20-initiated and caspase-8-targeted mechanism by which HBx modulates apoptotic signaling and increases hepatic susceptibility to the damaging agent, which might provide novel insight into HBV-related liver pathology.

## Introduction

Liver failure caused by hepatocyte death and tissue damage is one of the leading causes of HBV-related liver diseases [1]. It has been demonstrated that HBV infection can cause necrosis and apoptosis in liver cells, but the underlying mechanism remains largely elusive [2, 3]. Evidence has shown that hepatocyte apoptosis during HBV infection was essentially mediated by effector molecules, such as tumor necrosis factor (TNF), Fas ligand (FasL) and TNF-related apoptosis-inducing ligand (TRAIL), which were shown to be highly expressed in patients with HBV infection [4, 5]. Among them, TRAIL was distinguished by its ability to preferentially induce apoptosis in cancer and virus-infected cells but not normal cells [6]. Upon binding to death receptor (DR)4 and/or DR5, TRAIL causes the recruitment of caspase-8 and receptor-interacting protein (RIP)1 via Fas-associated death domain (FADD), forming the death-inducing signaling complex (DISC) and thereby initiating apoptotic signaling [7]. Additionally, TRAIL was found to be involved in the inflammatory responses in HBV-infected liver cells, which may further exaggerate the liver immunopathology. Of clinical significance, the amount of TRAIL was shown to be correlated with the extent of liver injury in HBV infection, particularly in patients with chronic hepatitis B (CHB) [5, 8].

HBx is a virally encoded protein that plays a key role in HBV-initiated biological processes, including viral replication, gene integration, tissue damage and cellular transformation. It has been shown that HBx can interact with host factors and modulate the apoptotic response in hepatic cells [9, 10]. Previous studies indicated that HBx can promote apoptotic signaling by increasing the expression and sustainability of key signaling molecules, such as Bax, Mcl1, and Bcl-2, or by triggering cytosolic calcium signaling in liver cells [11–13]. Other investigations, however, have argued that HBx exerted an inhibitory effect on the apoptotic response and facilitated the survival and proliferation of liver cells, which may contribute to hepatocellular carcinogenesis [14, 15]. Thus, the exact role of HBx in hepatic cell death remains controversial, and the related mechanism needs to be further established.

MicroRNAs (miRNAs) are a class of naturally occurring, small non-coding RNA molecules that are critically involved in a wide spectrum of fundamental cellular activities, ranging from proliferation, differentiation, and apoptosis to carcinogenesis. Recent data has demonstrated that miRNA can participate in virus-host interactions and exert regulatory effects on the process and outcome of viral infection [16]. Several miRNAs have been identified as specifically induced by HBV or viral components, thereby modulating hepatocyte behavior and liver physiopathology [17]. MiR-125a is one of these miRNA proven to play an essential role in the pathogenesis of HBV-associated liver disorders. It has been found that in HBsAg/anti-HBe-positive patients, the levels of liver miR-125a correlated with the HBV load and thus reflected the severity of liver disease [18]. Further study indicated that miR-125a can enhance viral replication by directly targeting and activating the viral gene sequence [19]. It also inhibited the proliferation and metastasis of hepatocellular carcinoma, presumably by repressing matrix metalloproteinase (MMP) 11, sirtuin 7 and cyclin D1 [20, 21]. Thus, the importance of miR-125a in HBV pathology has recently attracted attention, but its role in hepatocyte damage has not been explored.

A20, also known as TNF alpha inducible protein (TNFAIP) 3, is an E3 ubiquitin ligase and a key regulator of fundamental biological processes, such as immunity, inflammation and apoptosis [22, 23]. Because polyubiquitin conjugation to apoptotic molecules has emerged as an additional layer of apoptotic regulation [24], the importance of A20 in modulating cell death has now been increasingly recognized. Recent data showed that A20 impeded the apoptotic response by promoting the ubiquitination of RIP1, thereby blocking formation of the DISC complex [25]. These data suggest that A20 might have a regulatory effect on apoptotic signaling via its ubiquitinase activity, but its mode of action remains to be confirmed.

In the present study, we provide compelling data to show that HBx can induce the expression of A20 through the upregulation of miR-125a, which in turn promotes TRAIL-induced hepatocyte apoptosis. Remarkably, we demonstrate that HBx potentially inhibited the K63-linked ubiquitination of caspase-8 by downregulating A20 levels, enhancing caspase-8 cleavage, DISC formation and, hence, death signaling. These findings will surely extend our mechanistic understanding of hepatic cell death and liver pathology during HBV infection.

## Materials and Methods

### Reagents and antibodies

TRAIL was purchased from PeproTech, Inc. (Rocky Hill, NJ, USA). The HBx-specific antibody was purchased from Abcam, and the anti-RIP1 antibody was purchased from BD Bioscience (Franklin Lakes, NJ, USA). The antibodies against A20, caspase-3, cleaved caspase-3, caspase-8, caspase-9, caspase-7, PARP, DR5, FADD, and K63 ubiquitin were all obtained from Cell Signaling Technology (Beverly, MA, USA). The anti-β-actin antibody was from Sigma (St. Louis, MO). The horseradish peroxidase-conjugated secondary antibody was purchased from Santa Cruz Biotechnology (Santa Cruz, CA). The protein A/G plus agarose beads were purchased from Santa Cruz Biotechnology (Santa Cruz, CA).

### Plasmid constructions

The mammalian expression plasmid for Flag-tagged A20 and its mutants were constructed by the insertion of PCR-amplified human A20 or its mutant cDNAs into a pCMV-Tag2B vector (Promega). The miRNA reporter constructs were generated as previously described [26]. Briefly, the fragment containing the putative miR-125a binding site, including either the coding-region fragment of A20 ORF (1680-2096bp) or the 3′-UTR (348-635bp), was generated by PCR and cloned into the pMIR-report vector (Ambion). Site-directed mutagenesis was applied to change the six nucleotides within the seed sequence. The mammalian expression plasmid for HBx was previously described [27]. All constructs were confirmed by DNA sequencing.

### Cell culture and transfection

The immortalized liver (L-O2) and HepG2 cell lines were maintained in RPMI-1640 medium or Dulbecco’s modified Eagle’s medium (DMEM) supplemented with 10% fetal calf serum and 100 U/mL penicillin and streptomycin. To construct the cell line stably expressing HBx, L-O2 cells were transfected with 2 μg pCMV-Tag2B empty vector or pCMV-HBx using X-treme DNA transfection HP (Roche). G418 was added to the medium at a final concentration of 600 μg/ml. The cell lines stably transfected with pCMV-HBx (termed L-O2-HBx cells) or pCMV-Tag2B empty vector (termed L-O2-pCMV cells) were analyzed by PCR.

### MiRNA and RNA interference

The miR-125a mimic, miR-125a inhibitor and their non-specific controls, HBx-targeted siRNA, RIP1-targeted siRNA and non-specific siRNA, were synthesized and purified by Gima (Shanghai). RNA oligonucleotides were transfected using the X-tremeGENE siRNA Transfection Reagent (Roche).

### 5-Aza-2′-deoxycytidine treatment and methylation analysis

L-O2 and L-O2-HBx cells were treated with the DNA methylation inhibitor 5-Aza-2′-deoxycytidine (Aza; Sigma-Aldrich) at a final concentration of 5 μM. After 72 h, the cells were harvested, and RNA was extracted for qRT-PCR analysis. Genomic DNA (2 μg) from L-O2 and L-O2-HBx cells was modified with sodium bisulfite using EpiTect Bisulfite Kits (QIAGEN). PCR and bisulfite sequencing analysis were performed as previously described [28]. Amplified bisulfite sequencing PCR products were cloned into the pMD-18T vector (Takara) and sequenced.

### Quantitative real-time polymerase chain reaction (qRT-PCR) and reverse transcription-PCR (RT-PCR)

Total RNA was extracted from the cells (or tissues) using TRIzol (Invitrogen, Carlsbad, CA, USA) according to the manufacturer’s protocol. For miR-125a-5p quantitative PCR, cDNA was synthesized with TaqMan MicroRNA hsa-miR-125a-5p specific primers (Applied Biosystems) using the TaqMan MicroRNA Reverse Transcription kit (Applied Biosystems). qRT-PCR was performed in duplicate using QuantiTect SYBR Green PCR Master Mix (Applied Biosystems, Foster City, CA) and analyzed on an ABI Prism 7500 analyzer (Applied Biosystems, Foster City, CA). The primers used were as follows: A20, forward, 5′-AAAGCCCTCATCGACAGAAA -3′, reverse, 5′-CAGTTGCCAGCGGAATTTA-3′; TRAIL, forward, 5′-accaacgagctgaagcagat-3′, reverse, 5′-cagcaggggctgttcatact-3′; and GAPDH, forward, 5′-GGAGTCAACGGATTTGGT-3′, reverse, 5′-GTGATGGGATTTCCATTGAT-3′.

### Luciferase reporter gene assays

Luciferase reporter gene assays were performed using the Dual-Luciferase Reporter Assay System (Promega, Madison, WI, USA). Briefly, cells were cotransfected with miR-125a and the reporter constructs containing the intact or the mutant seed sequence of A20. After 24 h, the cells were harvested and assayed for firefly and Renilla luciferase activities.

### Analysis of apoptosis by Annexin V staining and the TUNEL assay

To evaluate apoptosis, L-O2 or L-O2-HBx cells were stained with Annexin V and propidium iodide (PI) using a Vybrant Apoptosis Assay kit (Invitrogen, Carlsbad, CA) as previously described [10]. Alternatively, a DNA Fragmentation Detection kit with Fluorescent-TdT Enzyme (Calbiochem, San Diego, CA) was used to detect DNA fragmentation according to the manufacturer’s specifications.

### Analysis of Surface Expression of TRAIL Receptors

1 × 10^6^ cells were washed with FACS buffer (0.5% BSA/PBS), and then incubated with 2 μg anti-DR4 or anti-DR5 antibodies for 1 hour at room temperature. Cells were washed twice in FACS buffer and stained with FITC conjugated anti-rabbit Ig antibody (BD Pharmingen) for 30 minutes at 4°C. After washing twice, cells were subjected to florescence-activated cell sorting (FACS) analysis for Surface expression of DR4 and DR5 (Becton Dickinson Immunocytometry System, San Jose, CA).

### Immunoprecipitation and Western Blot Analysis

For immunoprecipitations (IPs), whole-cell extracts from 10^7^ cells were collected and lysed in IP buffer containing 1% (vol/vol) Nonidet P-40, 50 mM Tris-HCl (pH 7.4), 50 mM EDTA, 150 mM NaCl, and a protease inhibitor cocktail (Merck). After centrifugation for 10 min at 14,000 rpm, supernatants were collected and incubated with protein A/G plus agarose beads and 5 μg antibodies. After incubation overnight at 4°C, the beads were washed five times with IP buffer. Immunoprecipitates were eluted by 1% (wt/vol) SDS sample buffer. To conduct two-step immunoprecipitation, the first immunoprecipitation was dissociated in a PBS buffer containing 1% SDS and boiled for 10 min, followed by a 20-folds dilution in lysis buffer. The second immunoprecipitation was then performed using anti-K63-Ub Ab. The samples were finally resolved by SDS-PAGE and immunoblotted for caspase-8. For western blot analysis, immunoprecipitates or whole-cell lysates were subjected to SDS-PAGE, transferred onto nitrocellulose membranes, and blotted as previously described [29].

### In Vitro Ubiquitination analysis

In vitro ubiquitination was performed by using a ubiquitinylation kit (Enzo), the reaction was performed in a 50μl reaction volume containing 2.5μM biotinylated Ub, 100nM E1, 1μM UBC13 or UBCH5a, 5mM Mg-ATP, 100nM E3 and 2ul 10X reaction buffer. After 2 h of incubation at 37°C, reactions were terminated by adding SDS loading buffer and examined by HRP conjugated streptavidin by Western-Blot.

### Statistical Analysis

All data from three experiments are presented as the means ± SEM. Statistical significance was determined using a two-tailed Student’s *t*-test and indicated as *P < 0.05, **P < 0.01.

## Results

### A20 is inhibited by HBx in hepatocytes

**Fig 1:**
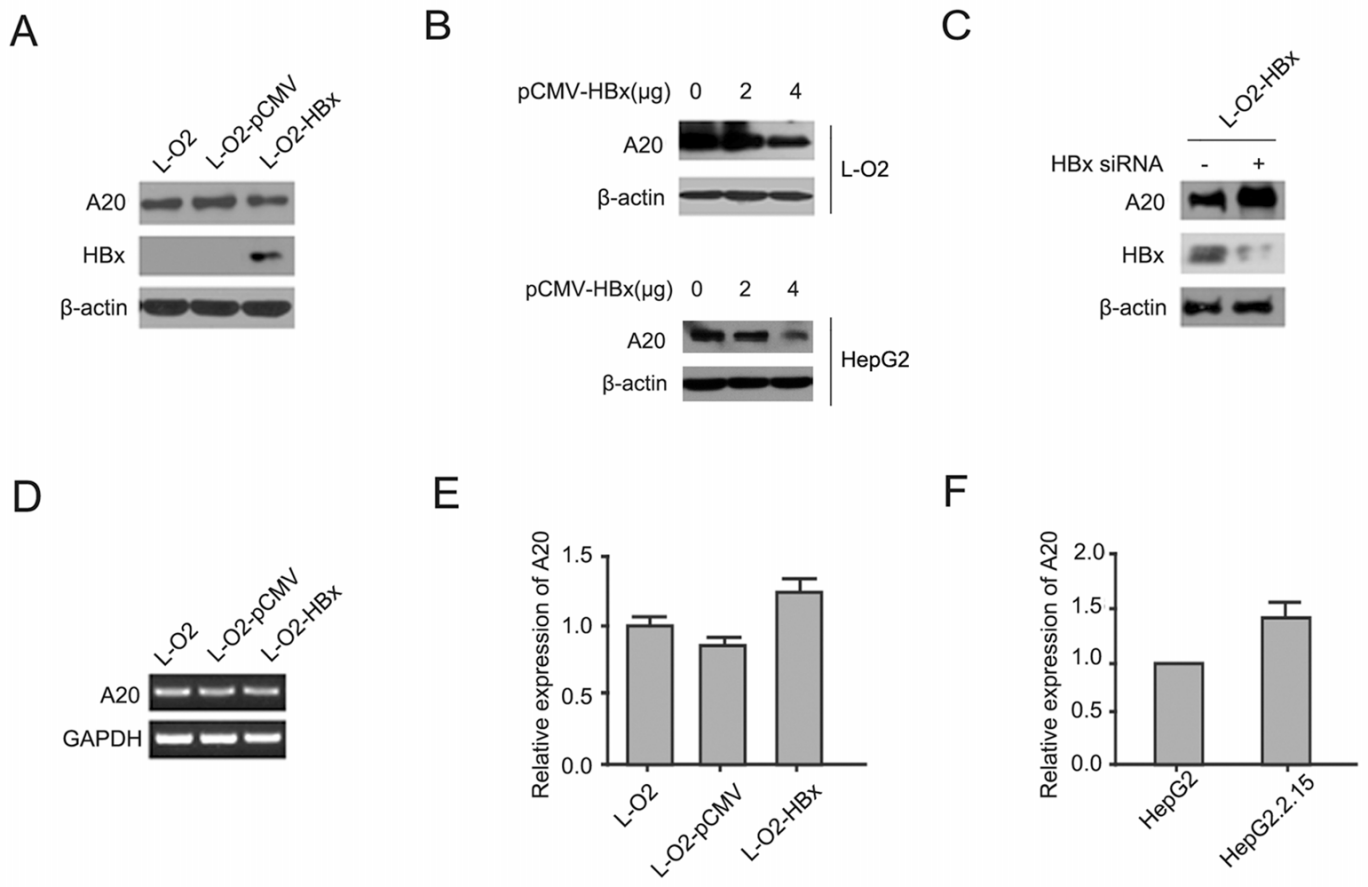
HBx inhibits A20 expression in hepatocytes at the protein but not the mRNA level. (A) L-O2, L-O2-pCMV, and L-O2-HBx cells were analyzed for A20 and HBx expression by western blot. (B) L-O2 cells and HepG2 cells were transfected with the pCMV-HBx plasmid at the indicated dose, and after 48 h, A20 expression was analyzed by western blot. (C) L-O2-HBx cells were transfected with control siRNA or HBx siRNA, and A20 and HBx expression was analyzed by western blot. (D, E) L-O2, L-O2-pCMV, and L-O2-HBx cells were analyzed for A20 mRNA expression by RT-PCR and real-time PCR. (F) HepG2 and HepG2.2.15 cells were analyzed for A20 mRNA expression by quantitative PCR. The results are from three independent experiments and are presented as the mean ± SEM.

To analyze the effect of HBx on the expression of A20 in liver cells, we first compared the expression of A20 in L-O2 cells with or without HBx overexpression. The result indicated that L-O2-HBx cells expressed slightly lower levels of A20 relative to L-O2 or L-O2-pCMV cells. Moreover, HBx was shown to repress the expression of A20 in a dose-dependent manner in L-O2 cells and HepG2 cells (Fig 1A and 1B). Consistently, deletion of HBx in L-O2-HBx cells appeared to derepress the expression of A20 (Fig 1C). However, it was noted that the mRNA level of A20 was not affected by HBx overexpression in hepatocytes according to RT-PCR and qRT-PCR analysis (Fig 1D and 1E). Likewise, no discernable difference in A20 mRNA levels was observed in the hepatoma HepG2 cells and the HBX-expressing HepG2.2.15 cells (Fig 1F). Thus, these data suggested that HBx may inhibit the expression of the A20 gene in hepatocytes at the post-transcriptional level.

### HBx upregulates miR-125a expression in hepatocytes

**Fig 2:**
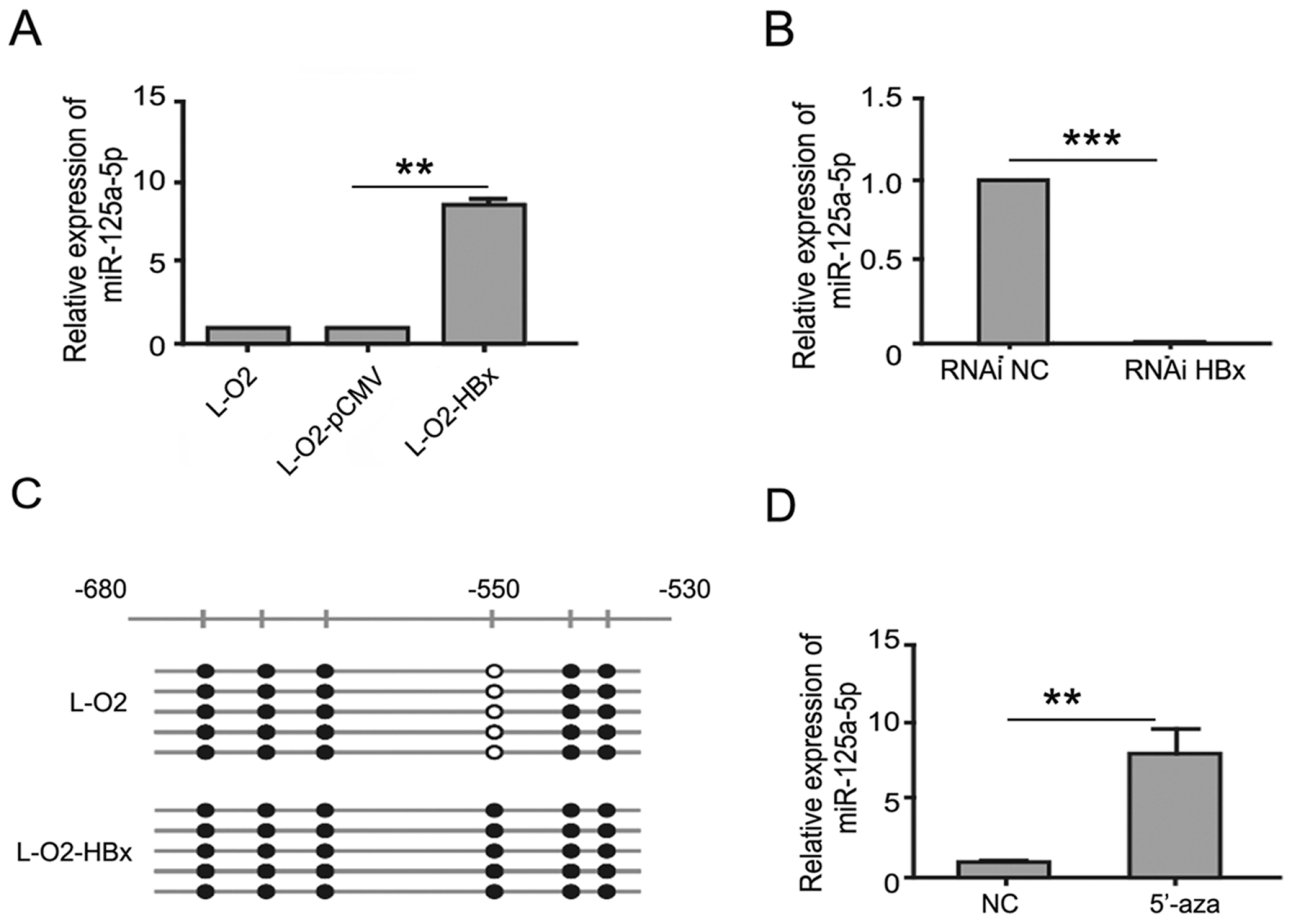
HBx regulates miR-125a transcription in a methylation-dependent manner. (A) L-O2, L-O2-pCMV, and L-O2-HBx cells were analyzed for miR-125a expression by real-time RT-PCR. (B) L-O2-HBx cells were transfected with control siRNA or HBx siRNA, and miR-125a expression was analyzed by real-time RT-PCR. (C) The methylation of miR-125a CpG sites was analyzed in L-O2 and L-O2-HBx cells by bisulfite sequencing analysis. At least five independent clones were sequenced per sample. Open and filled circles represent nonmethylated and methylated CpG sites, respectively. (D) L-O2 cells were analyzed for miR-125a expression by qRT-PCR after treatment with 5 μM 5′-Aza for 72 h. Data are from three independent experiments and are presented as the mean ± SEM (**P<0.01, ***P<0.001).

We next sought to understand the mechanism by which HBx mediated the repression of A20 and focused on miR-125a, a presumed A20-targeted microRNA and, more importantly, a key player in liver physiopathology [19, 30]. We first examined the level of miR-125a in L-O2, L-O2-pCMV, and L-O2-HBx cells. The data showed that HBx overexpression in L-O2 cells led to a remarkable augmentation in miR-125a transcription, whereas HBx knockdown in L-O2-HBx cells abrogated the upregulation of miR-125a (Fig 2A and 2B).

Because HBx has been demonstrated to interact with DNA methyltransferase (DNMT) and induce the hypomethylation of distal intragenic CpG islands required for active expression [31, 32], we wondered whether methylation was involved in the HBx-mediated regulation of miR-125a. To this end, we analyzed the miR-125a promoter using CpG Island Searcher (http://www.ualberta.ca/stothard/javascript/cpg_islands.html, University of Alberta, Edmonton, Canada) and identified a classic CpG island (nucleotides -680 to -530) within its 5’ UTR region. Furthermore, using bisulfite PCR sequencing, we demonstrated that the -550 CpG site was highly methylated in L-O2 but not L-O2-HBx cells (Fig 2C). These data indicated that HBx may trigger the expression of miR-125a by interfering with the methylation of its promoter. In support of this interpretation, when the DNA methylation inhibitor 5-Aza-2′-deoxycytidine was applied to L-O2 cells, the suppressive effect of methylation was abrogated, and miR-125a was dramatically induced (Fig 2D).

### MiR-125a targets A20 in HBx-expressing hepatocytes

**Fig 3:**
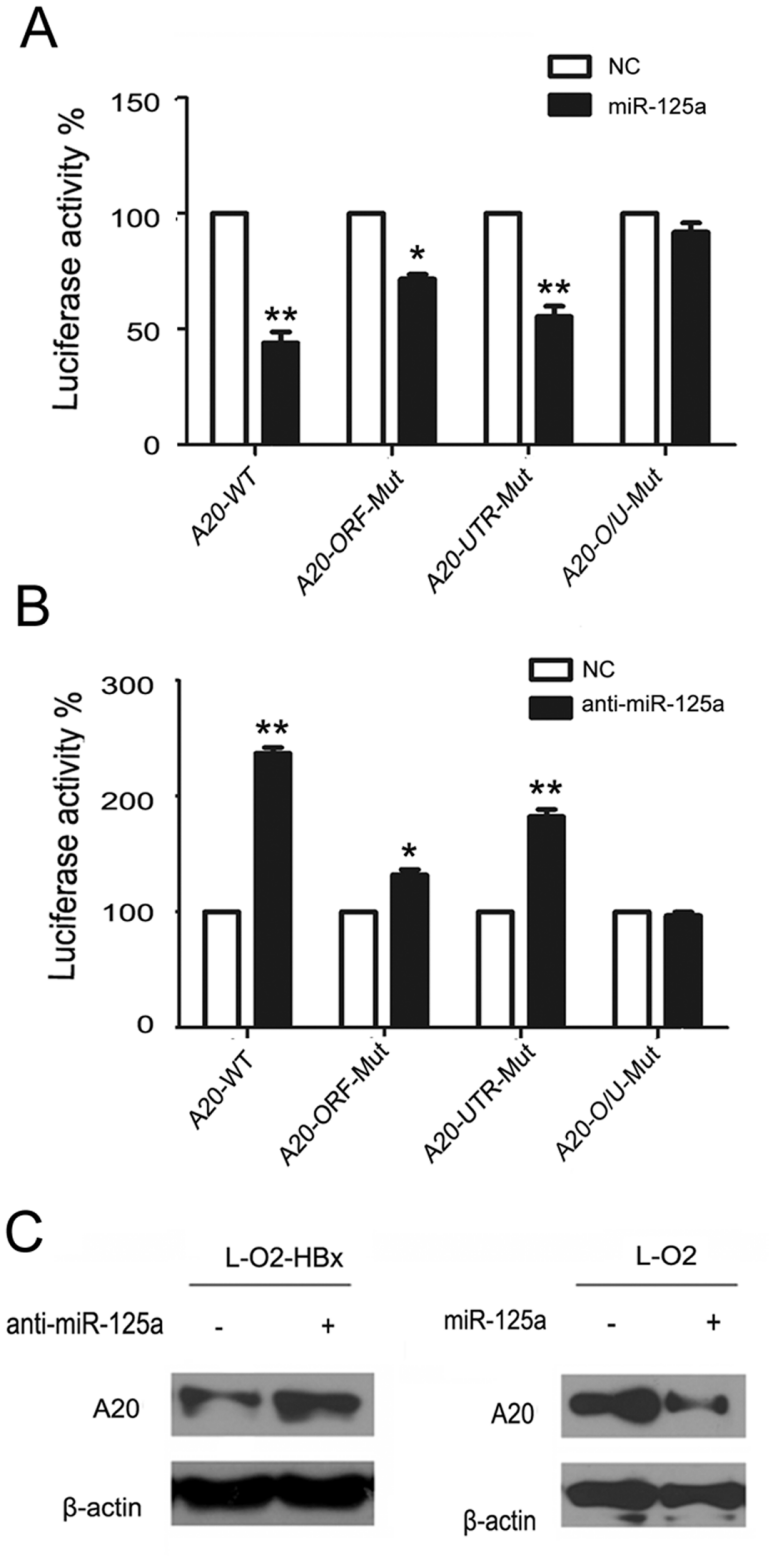
HBx inhibits A20 expression by upregulating miR-125a. (A) The luciferase activity was measured in 293T cells transfected with miR-125a or the control plasmid (NC), along with reporter plasmids (pMIR-) containing the intact or mutant binding sites at the A20 ORF or UTR. (B) Luciferase activity was measured in 293T cells transfected with the miR-125a inhibitor or the control plasmid (NC), along with the pMIR- constructs as in A. (C) The expression of A20 was detected by western blot in L-O2 cells transfected with miR-125a, anti-miR-125a or their controls. Data are from three independent experiments and are presented as the mean ± SEM. *P<0.05, **P<0.01 compared with the NC controls.

The observed inverse correlation between miR-125a and A20 levels in HBx-expressing L-O2 cells prompted us to hypothesize that A20 might be directly targeted by miR-125a and involved in the action of HBx. To test this hypothesis, we first analyzed the direct binding of the A20 sequence by miR-125a using the reporter system. Because both the ORF (1680–2096 bp) and 3′UTR (348–635 bp) within the A20 gene have been reported to possess the miR-125a seed sequence [26], we constructed the reporter plasmids containing the A20 ORF and 3′-UTR, as well as the corresponding mutants with a deletion at the binding site. The result showed that the reporter activity of the intact plasmid was significantly decreased by miR-125a compared with the NC control. However, the effect of miR-125a was affected by a mutation at the ORF or the 3′-UTR of A20 and was completely abrogated when both binding sequences were mutated (Fig 3A). Consistently, the inhibition of miR-125a expression caused a profound increase in the reporter activity, although this effect was repressed by the mutant construct (Fig 3B). Moreover, A20 expression in L-O2-HBx cells was significantly enhanced upon miR-125a inhibition, whereas the enforced expression of miR-125a led to a lower level of A20 in L-O2 cells (Fig 3C), suggesting that the HBx-mediated upregulation of miR-125a might contribute to its repression of the A20 gene. Therefore, our findings indicated that HBx can drive the miR-125a-mediated regulation of A20 in hepatocytes.

### MiR-125a/A20 is responsible for the HBx-mediated modulation of hepatocyte apoptosis

**Fig 4:**
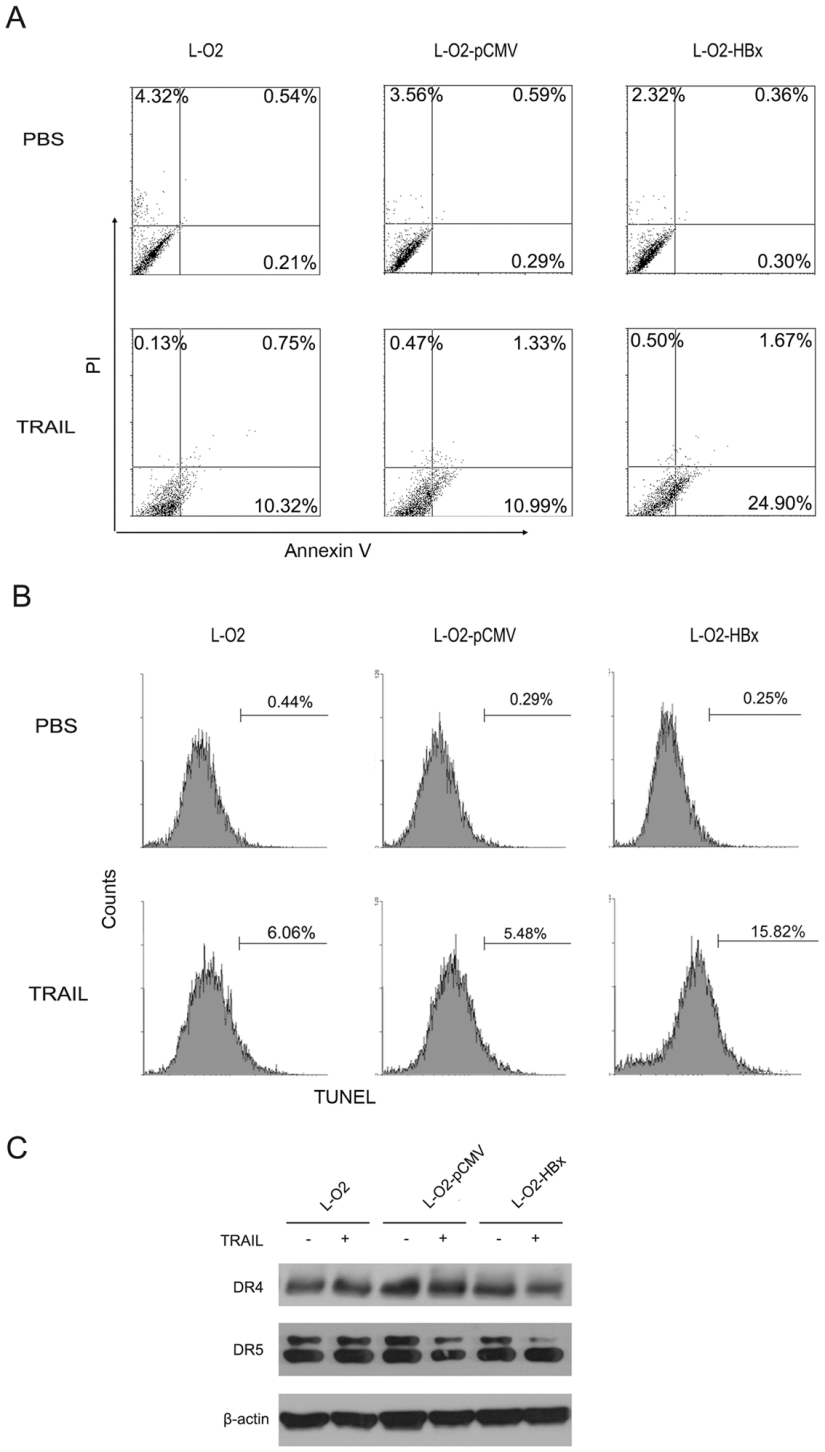
HBx enhances the sensitivity of hepatocytes to TRAIL-induced apoptosis. (A, B) L-O2, L-O2-pCMV, and L-O2-HBx cells were treated with PBS or 30 ng/ml TRAIL. After 24 h, the cells were labeled by Annexin V/PI (A) or TUNEL (B) and examined by flow cytometry.

Given the importance of HBx in regulating cell death and liver injury [11, 13], we wondered whether the miR-125a/A20 axis was involved in this process. We initially examined the susceptibility of hepatocytes to TRAIL-induced apoptosis with or without HBx. It was revealed that, compared with the parent L-O2 cells, L-O2-HBx cells were more vulnerable to TRAIL-induced death (Fig 4A and 4B). This augmented sensitivity did not appear to be related to the levels of the TRAIL receptors, as no difference in the expression of DR4 and DR5, the two receptors for TRAIL, was observed among L-O2, L-O2-pCMV and L-O2-HBx cells (Fig A and B in S1 File). Additionally, no discernable difference in surface expression of DR4 and DR5 was observed in L-O2 cells that were respectively transfected with miR-125a or negative control, and in L-O2-HBx that were respectively transfected with anti-miR-125a or negative control (Fig B in S1 File).

**Fig 5:**
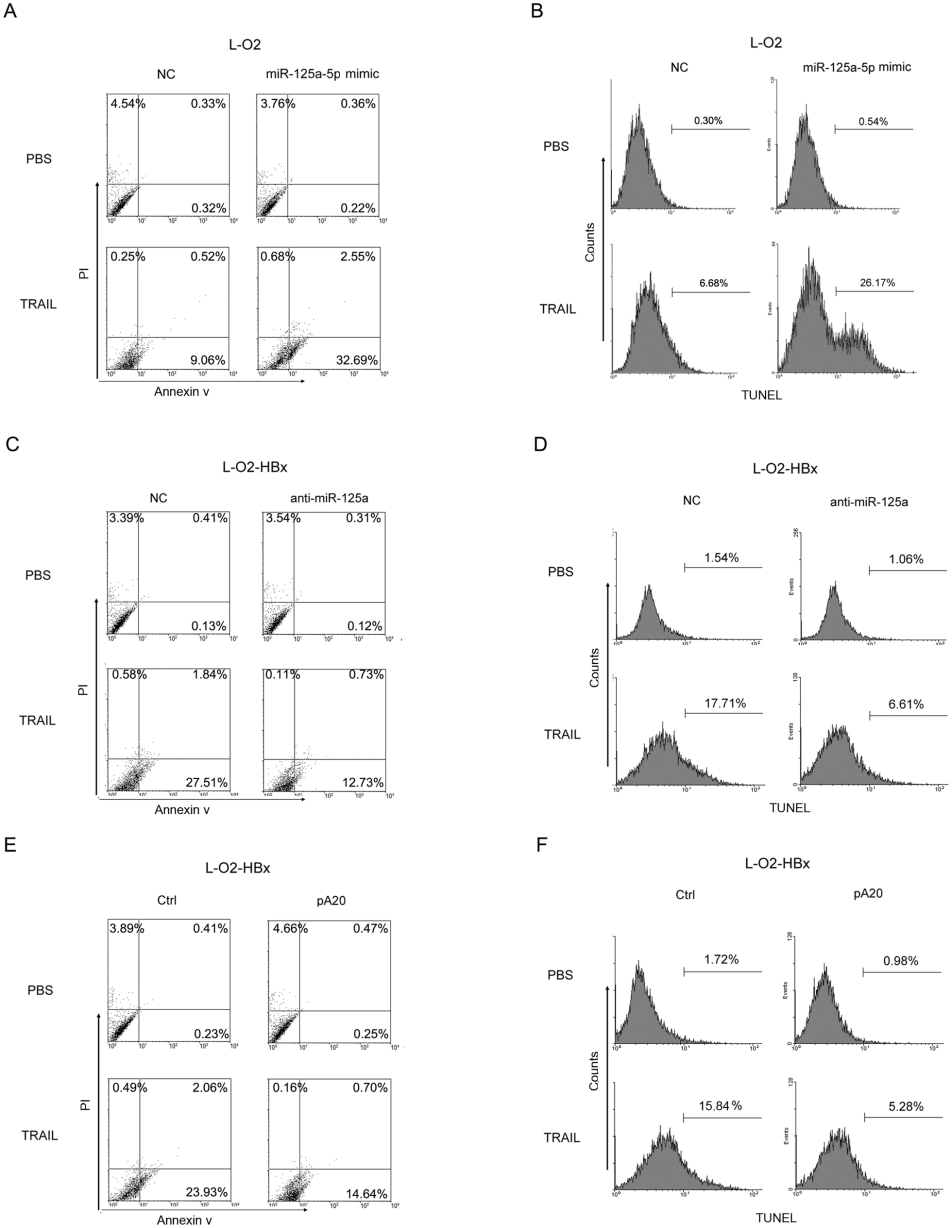
The transcript miR-125a/A20 mediates HBx-induced modulation of hepatocyte apoptosis. The apoptotic percentages were examined by flow cytometry 24 h after treatment with TRAIL (30 ng/ml) in (A, B) L-O2 cells transfected with miR-125a or the control, in (C, D) L-O2-HBx cells transfected with anti-miR-125a or the control, or in (E, F) L-O2-HBx cells transfected with the control plasmid or pCMV-A20. Representative data from at least three independent experiments are shown.

We then evaluated the role of miR-125a/A20 in the HBx-mediated modulation of cell death. As shown in Fig 5A and 5B, the overexpression of miR-125a in L-O2 cells generated a significant increase in the apoptotic population compared with cells transfected with the negative control. Conversely, the inhibition of miR-125a in L-O2-HBx cells caused a remarkable reduction in apoptosis (Fig 5C and 5D), and the ectopic expression of A20 had the same effect on these cells (Fig 5E and 5F), suggesting that interference in the miR-125a/A20 axis can override the ability of HBx to enhance TRAIL sensitivity. Together, our data identified a previously unknown role for miR-125a/A20 in the HBx-mediated regulation of hepatic cell death.

### HBx modulates apoptotic signaling via the miR-125a/A20 axis

**Fig 6:**
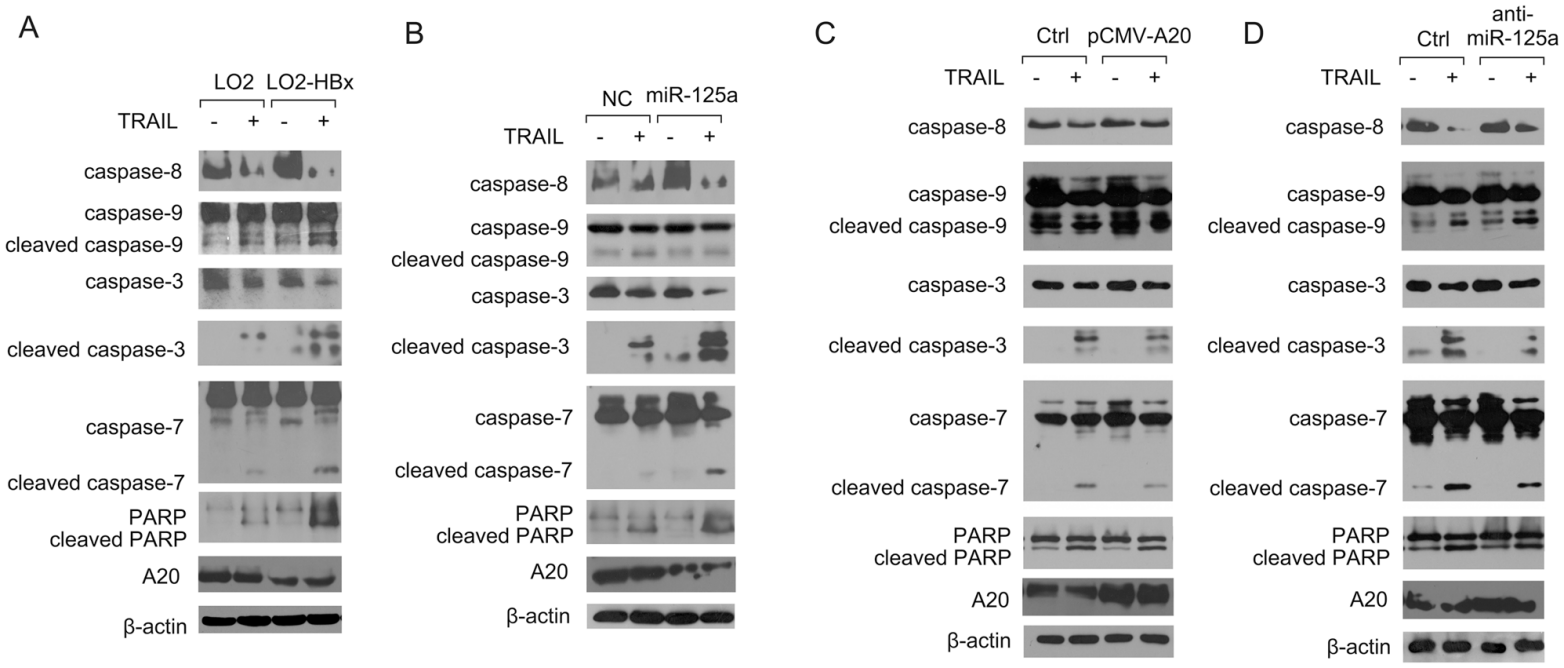
MiR-125a/A20 regulates TRAIL-induced apoptotic signaling in hepatocytes. The levels of caspases 8, 9, 3, and 7 and PARP were measured by western blot 24 h after TRAIL (30 ng/ml) treatment in (A) L-O2 and L-O2-HBx cells, in (B) L-O2 cells transfected with miR-125a or the negative control, in (C) L-O2-HBx cells transfected with the control plasmid or pCMV-A20, or in (D) L-O2-HBx cells transfected with anti-miR-125a or the negative control. Representative data from at least three independent experiments are shown.

To further understand the molecular mechanism by which HBx modulated TRAIL susceptibility, we tested the activities of caspases 8, 9, 3, and 7 and poly (ADP-ribose) polymerase (PARP), which represent the major molecular events involved in apoptosis. The result demonstrated that the cleavage of pro-caspases 8, 9, 3, and 7 and PARP was significantly increased in L-O2-HBx cells compared with control cells (Fig 6A). Additionally, the enhanced cleavage of caspases 8, 3, and 7 and PARP was induced upon miR-125a treatment in L-O2 cells (Fig 6B). However, the introduction of anti-miR-125a or A20 into HBx-expressing cells resulted in a remarkable downregulation of caspases 8, 3, and 7 and PARP activation in response to TRAIL challenge (Fig 6C and 6D). These results indicated that by regulating the miR-125a/A20 axis, HBx promoted apoptotic signaling and enhanced the hepatic susceptibility to TRAIL. Notably, caspase-9 activation was marginally affected in these conditions, suggesting that caspase-8 might be the principal target of the HBx/miR-125a/A20 loop during hepatocyte apoptosis.

### HBx enhances caspase-8 activity through downregulation of its K63-linked polyubiquitination by A20

**Fig 7:**
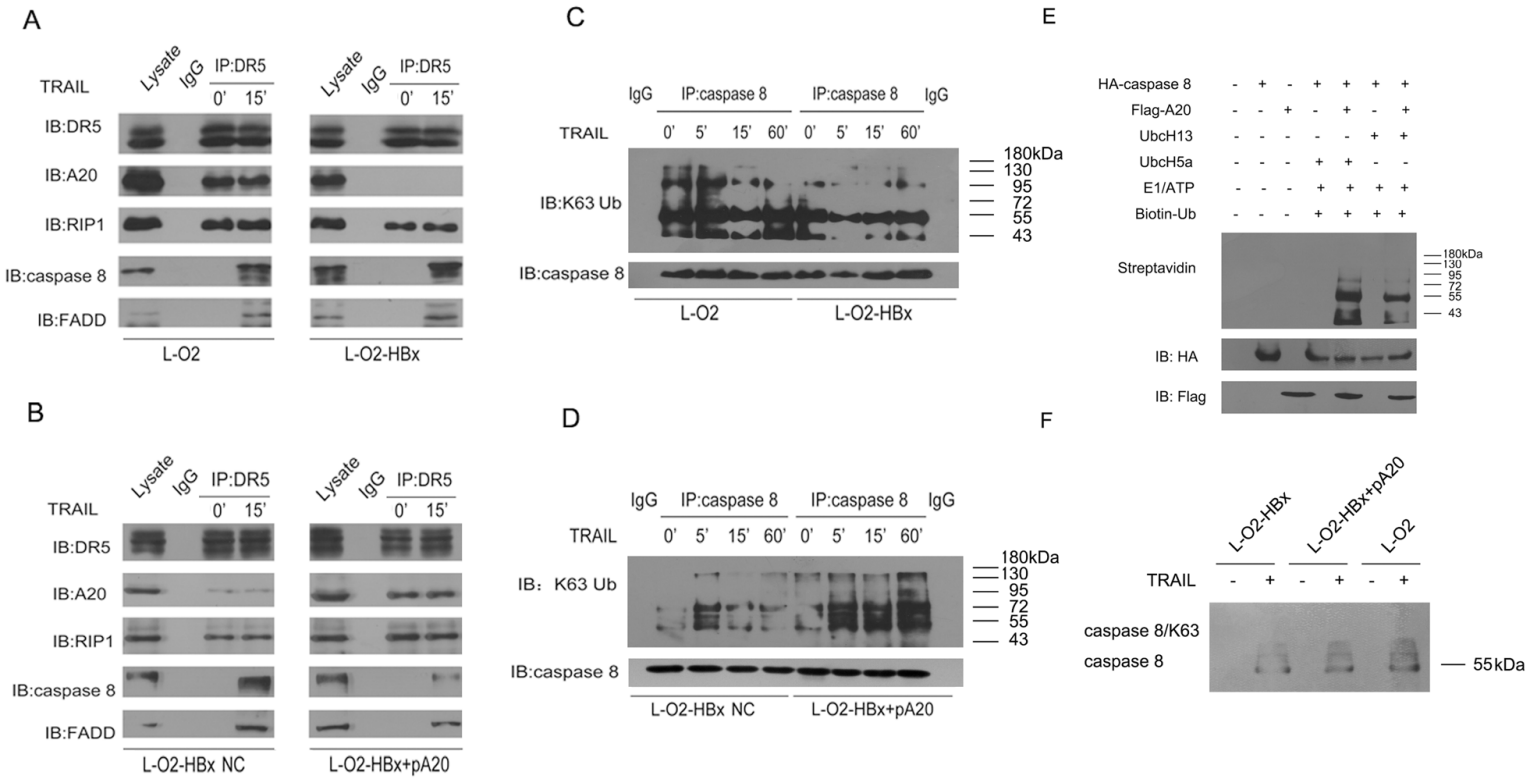
HBx enhances caspase-8 activity by downregulating its A20-mediated K63-linked polyubiquitination. (A, B) The interactions of DR5 with A20, RIP1, caspase-8, and FADD were detected by co-immunoprecipitation 15 min after treatment with TRAIL (30 ng/ml) in L-O2 and L-O2-HBx cells (A), or in L-O2-HBx cells transfected with the A20 expression vector or control vector (B). (C, D) K63-linked polyubiquitination of caspase-8 was detected after treatment with TRAIL (30 ng/ml) in L-O2 and L-O2-HBx cells (C) or in L-O2-HBx cells transfected with the A20 expression vector or control vector (D). (E) *In vitro* ubiquitination was carried out in a reaction consisting of the components as indicated (*top*) with ubiquitinated caspase 8 detected by a HRP linked streptavidin or specific antibodies on IB. (F) Caspase-8 was immunoprecipitated from L-O2, L-O2-HBx cells, or L-O2-HBx cells tranfected with pA20 upon TRAIL treatment, followed by a second immunoprecipitation with an anti-K63 ubiquitin antibody. The ubiquitination of caspase-8 was then detected by immunoblotting. Representative data from at least three independent experiments are shown.

It is well known that TRAIL initiates apoptotic signaling by binding to DR5, which subsequently causes the recruitment of caspase-8 via the FADD domain and, together with RIP1, formation of the DISC complex, thereby triggering apoptotic signaling [33]. Moreover, it is known that caspase-8 is subjected to E3 ubiquitin ligases and assumes an enhanced K63-polyubiquitinated inactive state [34, 35]. Therefore, we asked if A20, a prototypical E3 ligase, had a regulatory effect on caspase-8, thereby mediating the effect of HBx on apoptotic signaling. To this end, DISCs were isolated from L-O2 and L-O2-HBx cells following TRAIL treatment, and the signaling molecules involved were analyzed. The result showed that, although the presence of A20 and RIP1 in DISCs was slightly decreased in L-O2-HBx cells, the recruitment of FADD and caspase-8 was remarkably increased upon TRAIL treatment, as compared with that in L-O2 cells. Notably, caspase-8 had much lower levels of K63-linked ubiquitination, indicative of the deactivation, in L-O2-HBx cells compared with L-O2 cells (Fig 7A and 7B). Interestingly, we found that siRNA-mediated RIP1 knockdown caused a significant increase in TRAIL-triggered cellular death both in L-O2 and HepG2 cells, suggesting a protective role of RIP1 in hepatic damage (Fig A and B in S2 file).

Next, to examine if the ubiquitinase activity of A20 was responsible for the observed downregulation of caspase-8 polyubiquitination in HBx-expressing hepatocytes, we rescued the expression of A20 in L-O2-HBx cells and examined caspase-8 activity. Remarkably, the enforced expression of A20 largely abolished the HBx-induced repression of caspase-8 ubiquitination, thereby blocking its activation. Accordingly, the recruitment of FADD and caspase-8 to the DISC was inhibited (Fig 7C and 7D), and the apoptotic rate was reduced in the A20-expressing L-O2-HBx cells (Fig 5E and 5F). Furthermore, to substantiate that A20 can directly ubiquitinate caspase-8, we conducted in vitro ubiquitination assay. The results showed that caspase-8 could be ubiquitinated in the presence of A20 in an in vitro ubiquitination system containing biotin-UB, the K48-specific E2 enzyme UBCH5A or the K63-specific E2 enzyme UBC13, and ATP-Mg2+, indicating that A20 could ubiquitinate casepase-8 by facilitating the assembly of K48- or K63-linked polyUb chains. Moreover, two-step immunoprecipitation test indicated that TRAIL-induced K63-ubiquitination of caspase-8 was significantly decreased in L-O2-HBx cells relative to L-O2 cells. Restoration of A20 in L-O2-HBx cells however caused an increase in caspase-8 ubiquitination, further confirming the ubiquitinative effect of A20 on caspase-8 (Fig 7F). Thus, we conclude that HBx promoted TRAIL-initiated apoptotic signaling largely through the downregulation of A20 and the subsequent polyubiquitination of caspase-8.

## Discussion

In the present study, we demonstrated that HBx specifically induced the expression of miR-125a in hepatocytes, which in turn repressed the expression of A20. The functional significance of this regulation of miR-125a/A20 is revealed by the HBx-mediated increased sensitivity of hepatocytes to TRAIL-induced apoptosis. Mechanistically, the controlled expression of A20 by HBx was shown to potentially inhibit the K63-linked polyubiquitination of caspase-8, reciprocally enhancing the activation of caspase-8 and, hence, apoptotic signaling. These data reveal the pro-apoptotic effect of HBx in liver cells and provide novel mechanistic insight into HBV-associated cell death and liver damage.

Apoptosis is generally mediated through two pathways: the extrinsic and intrinsic pathways. It is known that TRAIL triggers the extrinsic pathway by engaging the death receptors DR4 or DR5 and requires FADD recruitment and DISC formation to initiate caspase-8-, and caspase-3-mediated signaling [33, 36]. Given the importance of TRAIL activity in the pathogenesis of liver pathology, much attention has been paid to the regulatory events targeting the involved apoptotic molecules. Recent data indicated that caspase-8, the key mediator of apoptotic signaling, can be modified by certain E3 ubiquitination ligases, such as HECTD3 and TRIM13 [34, 35]. These ligases have been shown to promote the K63-linked polyubiquitination of caspase-8, hindering its cleavage and activation and thereby inhibiting apoptotic signaling [25]. It appears that protein ubiquitination has emerged as a novel modulator of cell death, but we currently know little about this regulatory paradigm. In the present study, we identify the prominent role of miR-125a/A20 in HBx-mediated TRAIL susceptibility in hepatocytes, and, more importantly, we reveal an unknown role of A20 in modulating the apoptotic response.

A20 is a well-established E3 ligase featured as having both deubiquitinase and E3 ligase domains within its structure and is thus presumed to act as a ubiquitin-editing enzyme. The involvement of A20 in the apoptotic response has been revealed. A20 was shown to act downstream of TNFR1 and counteract the apoptotic effect of TNF by inactivation of the signaling molecule RIP1 [37]. Moreover, A20 was found to be preferentially expressed in hepatitis B virus (HBV)-related hepatocellular carcinoma (HCC) cells, contributing to cellular proliferation and survival [38, 39]. However, the molecular basis underlying A20-mediated regulation and its functional relevance to hepatic damage are still not completely understood. Herein, we provide compelling evidence to support the anti-apoptotic effect of A20 in liver cells, which is largely due to its ability to promote the polyubiquitination of caspase-8 and, hence, the inhibition of caspase-8 activation and its downstream signaling. Importantly, we show that the expression of A20 is prominently repressed by HBx in liver cells via miRNA-mediated regulatory machinery. These data delineate an unknown mechanism by which the HBx protein dictates cell fate in the liver, suggesting a potential molecular target for the prevention of HBV-associated pathologies. However, it should be noted that A20 is a ubiquitin-editing enzyme that can exert either deubiquitination activity via the ovarian tumor (OTU) domain at its N-terminal or E3 ligase activity via a zinc-finger domain at its C-terminal [38, 40]. Although the net effect of A20 during HBx-mediated apoptosis has been revealed in this study, functional dissection of the distinct domains of A20 might merit further investigation in an attempt to develop specific liver-protective agents.

It has been increasingly appreciated that miRNAs are critically involved in a wide range of pathological processes, including HBV-related liver disorders, but their relevance to hepatocyte death has not been fully explored. miR-125a is a liver-expressed miRNA that was previously shown to play a regulatory role in the proliferation of HBV-infected hepatocytes and HCC cells [18, 20]. Interestingly, almost simultaneously, we and the Potenza lab showed the specific induction of miR-125a by HBx in liver cells [41]. Nevertheless, to the best of our knowledge, our finding is the first report to link miR-125a to HBx-mediated TRAIL susceptibility in hepatocytes. Considering the correlation of TRAIL levels and liver diseases in the clinic, this finding surely provides a novel perspective on HBV-associated liver pathology. Remarkably, identification of the target molecule A20 and its modification of capase-8 activity expands our mechanistic understanding of the action of HBx. In addition, the present study demonstrates that the induction of miR-125a was largely due to the capability of HBx to disrupt hypermethylation of the miR-125 promoter, thus implicating the epigenetic network in HBx—associated hepatocyte behavior. Future studies will be conducted to address how HBx modulates DNA methylation at the miR-125a locus and whether there is any methyltransferase and/or demethylase involvement downstream of HBx or other viral antigens [42, 43].

In summary, we demonstrate herein that HBx sensitizes hepatocytes to TRAIL-induced apoptosis, and this effect occurs at least partly through the miR-125a/A20 axis. A20 plays a crucial role in counteracting apoptotic signaling via the modulation of caspase-8 ubiquitination and subsequent apoptotic signaling. These findings have delineated a novel biochemical mechanism underlying HBV-related cell death and hepatic failure.

## Supporting Information

S1 File HBx, miR-125a or anti-miR125a have no affects on the expression of DR4 or DR5. The level of DR4 and DR5 was detected by western blot in L-O2, L-O2-pCMV, and L-O2-HBx cells with or without 24 h treatment of TRAIL (30 ng/ml). Representative data from at least three independent experiments are shown (Fig A). The surface expression of DR4 and DR5 was detected by FACS in L-O2, L-O2-pCMV and L-O2-HBx cells, or in L-O2 cells transfected with miR-125a or the negative control, or in L-O2-HBx cells transfected with anti-miR-125a or the negative control, respectively. Representative data from at least three independent experiments are shown (Fig B).
(TIF)

S2 File Knockdown of RIP1 enhances the sensitivity of hepatocytes to TRAIL-induced apoptosis. The expression of RIP1 was detected by western blot in L-O2 and HepG2 cells transfected with RIP1 siRNA or the control (Fig A), and the apoptotic percentages in L-O2 or HepG2 cells were examined by flow cytometry 24 h after TRAIL (30 ng/ml) treatment (Fig B and C).
(TIF)
