# Supplementary figures and images for: Hepatitis B Virus X Protein Sensitizes TRAIL-Induced Hepatocyte Apoptosis by Inhibiting the E3 Ubiquitin Ligase A20
Source: PLoS One. 2015 May 20;10(5):e0127329. doi: 10.1371/journal.pone.0127329 (PMC4439114; doi:10.1371/journal.pone.0127329)

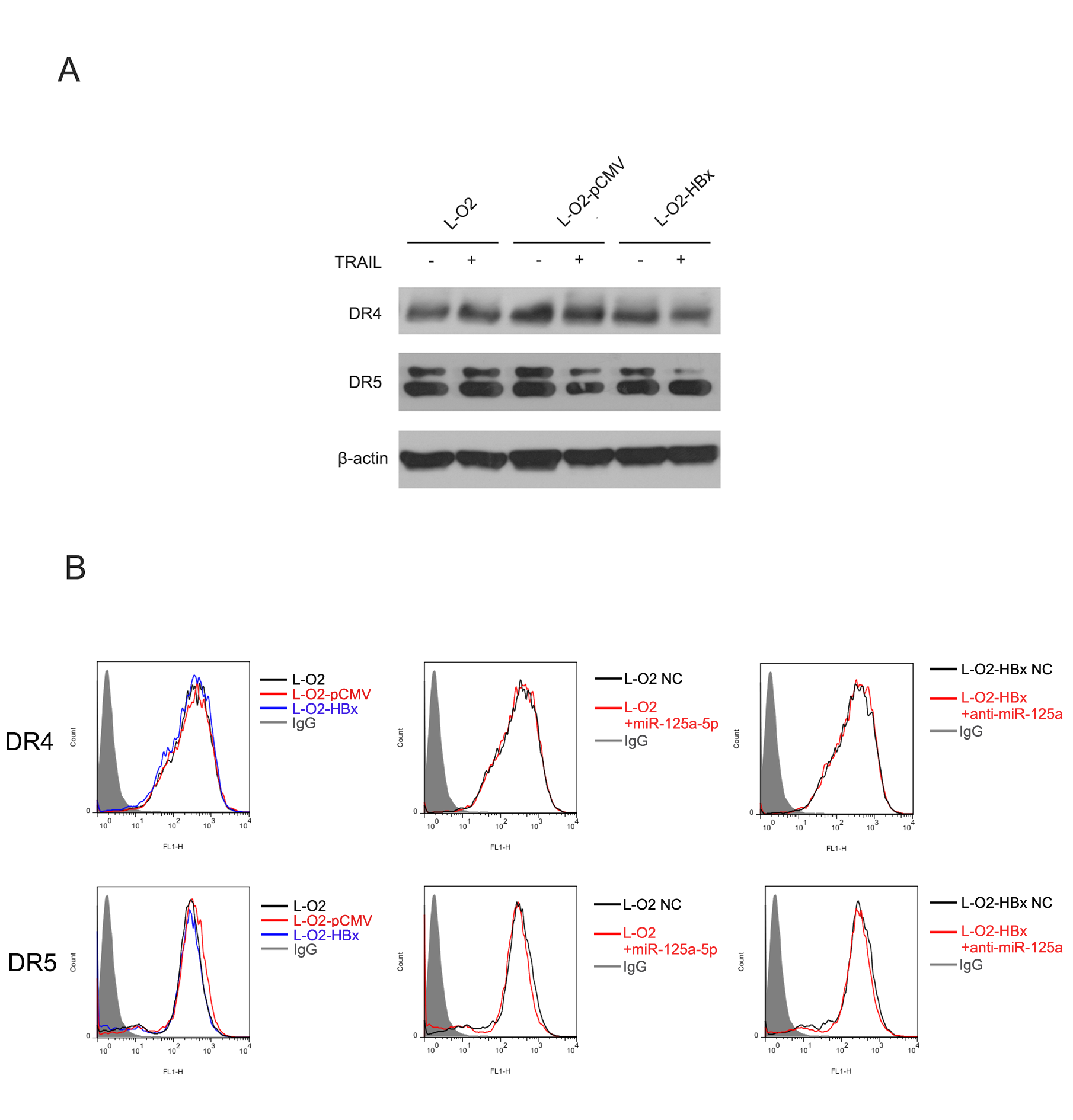

Supplement: S1 File — The level of DR4 and DR5 was detected by western blot in L-O2, L-O2-pCMV, and L-O2-HBx cells with or without 24 h treatment of TRAIL (30 ng/ml). Representative data from at least three independent experiments are shown (Fig A). The surface expression of DR4 and DR5 was detected by FACS in L-O2, L-O2-pCMV and L-O2-HBx cells, or in L-O2 cells transfected with miR-125a or the negative control, or in L-O2-HBx cells transfected with anti-miR-125a or the negative control, respectively. Representative data from at least three independent experiments are shown (Fig B). (TIF) [file pone.0127329.s001.tif]

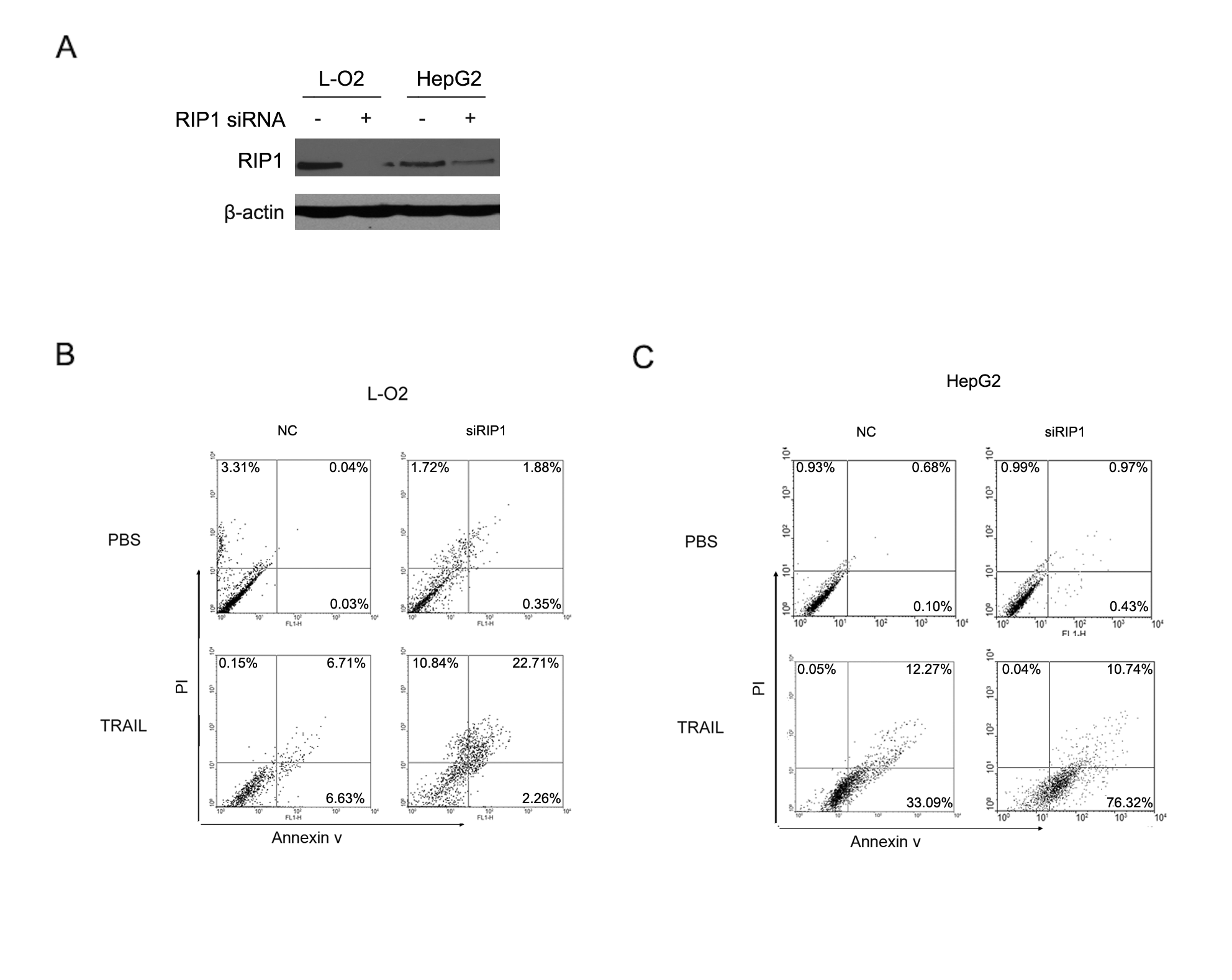

Supplement: S2 File — The expression of RIP1 was detected by western blot in L-O2 and HepG2 cells transfected with RIP1 siRNA or the control (Fig A), and the apoptotic percentages in L-O2 or HepG2 cells were examined by flow cytometry 24 h after TRAIL (30 ng/ml) treatment (Fig B and C). (TIF) [file pone.0127329.s002.tif]
